# Findings of a Community Health Needs Assessment Survey in Al Najaf Governorate Iraq: A Snapshot of Al Marashda’s Health Status

**DOI:** 10.7759/cureus.61935

**Published:** 2024-06-08

**Authors:** Hayder Madlool, Naseem Sadoon, Mohammed Kamel, Ali Mohammed, Ameer Hameed, Mohammed Haleem, Manoochehr Karami

**Affiliations:** 1 Department of Epidemiology, School of Public Health and Safety, Shahid Beheshti University of Medical Sciences, Tehran, IRN; 2 Department of Public Health, Najaf Health Directorate, Najaf, IRQ

**Keywords:** iraq, poverty, health priorities, community survey, air pollution

## Abstract

Background: Community health assessment (CHA) is a well-known method for identifying and analyzing community health needs. This CHA survey aimed to identify and analyze community health needs and assets to prioritize these needs and to plan and act upon significant unmet community health needs.

Methods: The CHA was planned based on the suggested standard of the North Carolina Guide including eight phases from July to December 2023. The CHA survey was performed among Al-Marashda region residents in the Al-Manathera district. The sample size of our study was 184 interviews of 12536 population. The primary data, which included demographic information, quality of life statements, and community improvement, were collected from the community using a questionnaire through opinion surveys and focus groups, while the secondary data which included the social, health, and economic status of Al-Marashda region residents were obtained from district and governorate sources. Analysis of whole data sources allowed 10 areas of community concern to be identified.

Results: Findings from the CHA survey showed that diabetes and high blood pressure, poverty and unemployment, and air pollution were the most common public health problems as priorities.

Conclusions: The high-priority problems of Al-Marashda are in common with the noncommunicable diseases (NCDs) priority in Al Najaf. However, poverty and air pollution are specific to the Al-Marashda region. Public health authorities and the city governorate are advised to consider, support, and develop community diagnosis documents to implement appropriate interventions.

## Introduction

The term “community health assessment” (CHA) is also known as “community health needs assessment” (CHNA) [1]. The definition for community needs health assessment is “a process to determine the health status, needs, and health resources in the county”. We use the term CHA to describe the relevant health needs of Al-Marashda area residents so that treatment plans can be developed accordingly. CHA is one of the core proficiencies for public health providers, giving them a better understanding of strengths and drawbacks [2].

CHA considers a systematic process including the community to recognize and analyze community health needs and assets to prioritize these needs and plan and act upon significant unmet community health needs [3]. The process of identifying unmet health needs in a population is critical for local authorities seeking to plan appropriate and effective programs to meet these needs [4]. It is not a ”one-off” activity but rather an ongoing developmental process. It can be modified over time, not only by looking for problems and needs but also by assessing the strengths and resources/assets that promote well-being in the community [5]. It is usually designed to evaluate gaps between existing situations and desired outcomes, along with possible solutions for addressing the gaps [4].

CHA is known to be an efficient tool for addressing the health needs of communities that may be unmet by health systems and other regional authorities. By its very nature, dissemination and implementation is well to foster community development [6-8]. Although Iraq has made progress in improving the general health status of its population over the past decade. For example, life expectancy is high and infant mortality rates are low - but they still lag behind other countries. In spite of violence has been the leading cause of death over the past decade, chronic diseases now represent 43% of all adult deaths, therefor CHA is needed now [9]. Accordingly, we performed a CHA survey among Al-Marashda, an unrepresented region, to identify and prioritize health needs using the North Carolina Guide.

## Materials and methods

**Figure 1:**
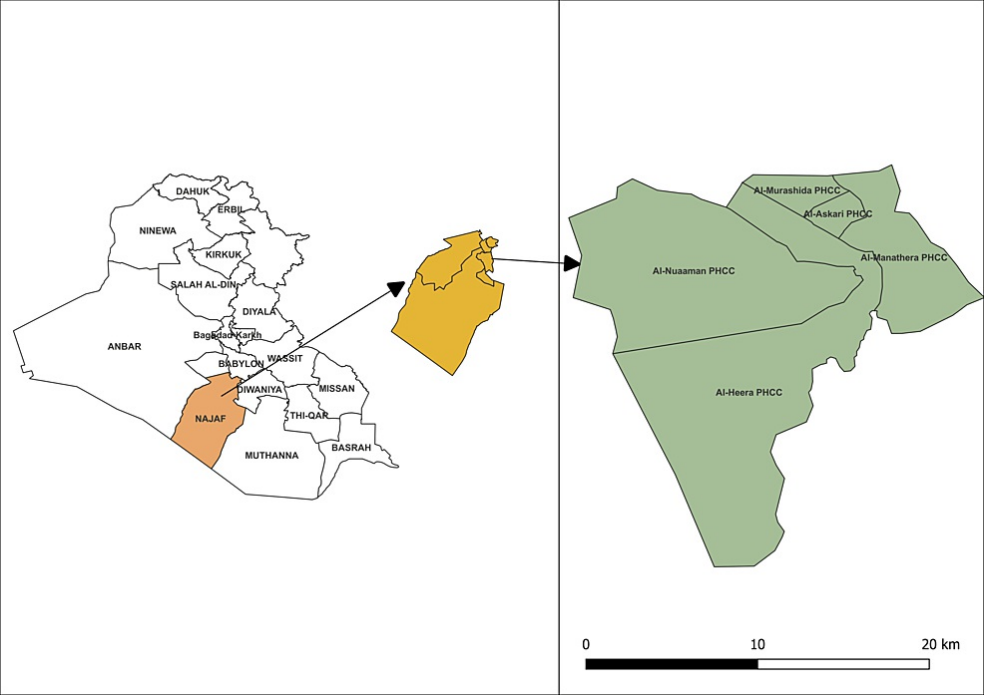
The geographical location of the study setting The figure was drawn by the authors of this article.

We have used the standard eight phases of the CHA survey based on the North Carolina Guide from July to December 2023 [10]. The setting of this CHA survey was Al-Marashda region residence in the Al-Manathera district. Al-Manathera district center is located approximately 18 km south and southwest of the Al-Najaf district center, covering an area of 76416.59 hectares (Figure 1) [11]. Al-Najaf Al-Ashraf is one of the holiest cities in Iraq, located in the southwest of Iraq, to the southeast of Baghdad, which is 160 km from the capital city of Iraq [12].

**Figure 2:**
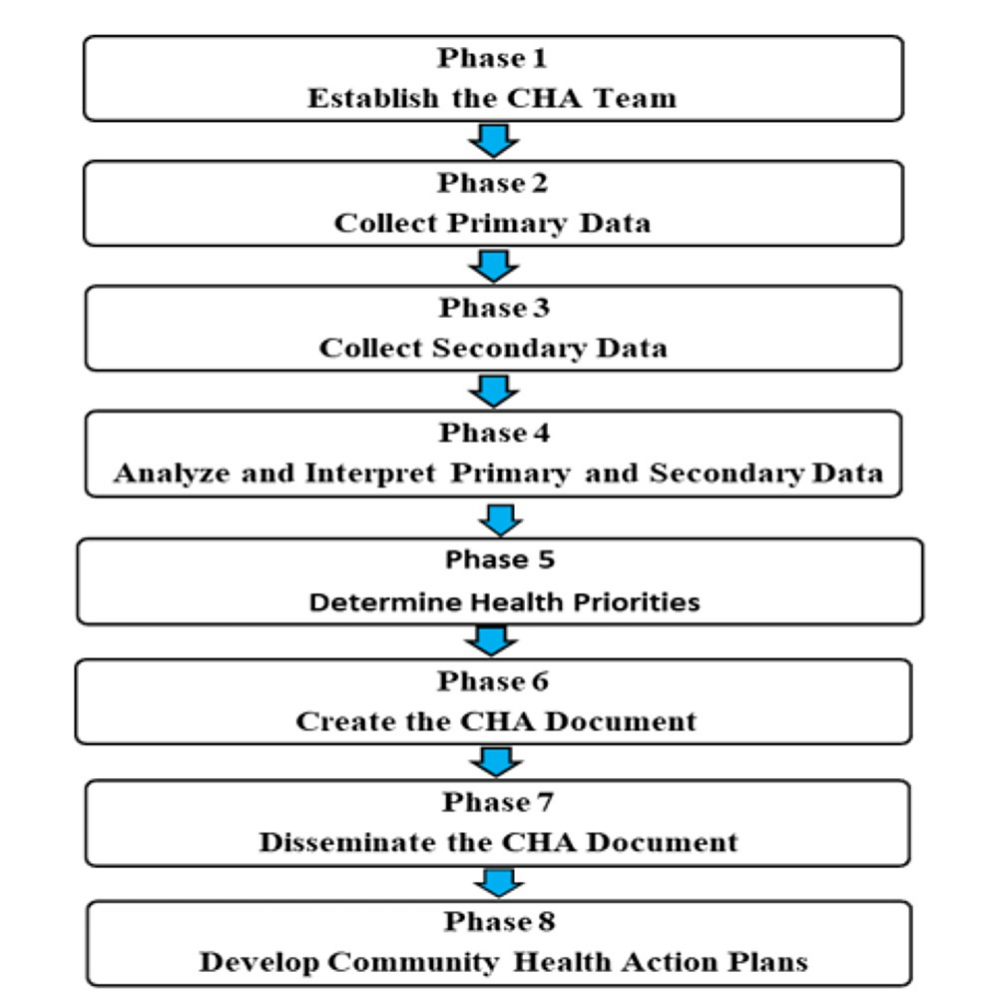
The community health assessment process according to North Carolina (Community Health Assessment Guidebook) The figure was drawn by the authors of this article.

According to the Community Health Assessment Guidebook (2014) in North Carolina, the CHA process includes eight phases (Figure 2). First, we will institute a CHA team. Second, primary and secondary data collection, followed by analyzing and interpreting these data, defining health priorities, making the CHA document, disseminating the CHA document, and finally developing the Community Health Action Plans [10].

Establish the CHA team

Under the leadership of the staff of the epidemiology section in the public health department, a CHA team was formed with members from different sections in the public health department and the Al-Marashda Primary Health Care Center (PHCC). The target goal of this team was to plan and implement CHNA in 2023.

Primary Data Collection

A community health opinion survey was developed, which included 39 questions on topics such as demographic information, quality of life statements, and community improvement. The data was collected from 20 to 26 August 2023. The interview locations were determined using a 2-stage cluster sampling method (three clusters were selected, and interviews were subsequently conducted via systematic random sampling). The sample size was calculated using Epi-Info version 5.5.13 statistical software according to the following parameters: 5% type one error and 90% power. According to a study conducted in Iran, the percentage of economics and employment (12.5%) has been reported for CHA [13]; hence, the sample size is 168. Then, by adding a 10% nonresponse rate, the final calculated sample size is 184. The data were electronically recorded at the time of the interview via mobile phone according to a questionnaire created by the Kobo Toolbox Program. The data were organized daily and analyzed by Microsoft Excel version 2019.

Three group discussions were conducted in the Al-Marashda region in September 2023 based on their demographics or their interest in talking about a health topic. These groups consisted of the following populations: healthcare workers, individuals suffering from chronic health conditions, and individuals with physical disabilities that substantially limit at least one essential life activity. The focus group meetings continued for one hour and included approximately six or ten members per group. Focus group members were informed about the general aim of the CHNA, the details of participation, the data collection detail to ensure confidentiality, and their rights as participants. Participants were asked to provide verbal consent to participate in the assessment.

Secondary Data Collection

Community health depends on a wide variety of factors, so data from different sources included (Al-Marashda PHCC, Al Najaf Health Directorate and Al Najaf Environment Directorate) need to be collected to obtain an overall picture of Al-Marashda’s health.

Other standard phases of the CHA survey including Descriptive Analysis and Interpretation of the Primary and Secondary Data Primary Data and Determination of Health Priorities were described in the results section.

## Results

Analysis and interpretation of the primary and secondary data

Primary Data

**Figure 3:**
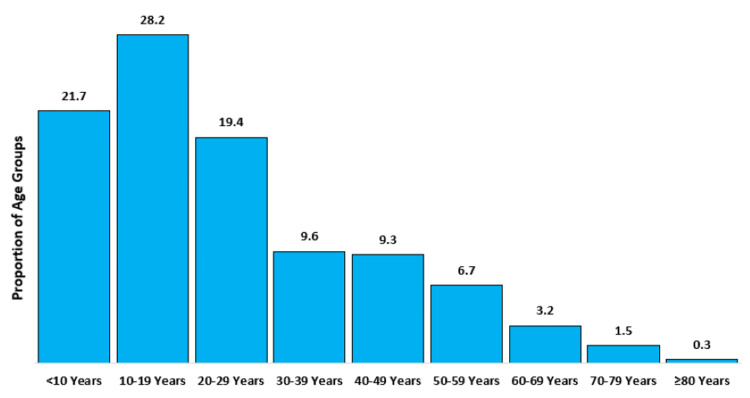
Distribution of age groups in the Al-Marashda region, Al-Manathera district The figure was drawn by the authors of this article.

Over a seven-day period, three teams carried out a total of 184 interviews (100% completion rate) in the Al-Marashda district. The mean number and standard deviation of family members was 7 ±2.7. The total number of family members was 1307, with a mean age of 24 ±17.5 years and a median of 20 years (IQR: 11,34). The highest age group was 10-19 years, accounting for 28.2% (n=369) of all family members Figure 3. Approximately 54% (n= 703) of family members were males, with a male-to-female ratio of 1.16:1. Regarding educational level, 50% (n= 92) of respondents had no formal education, followed by primary education 32% (n=58), secondary education 8% (n=14) and higher education 11% (n=20).

In the current survey, 66% (n= 121) of participants disagreed with the statement “There is plenty of economic opportunity in the Al-Marashda region”, and 63% (n= 115) of respondents disagreed with the statement “There are good and sufficient schools in the Al-Marashda region”. Most of the respondents say there are no Parks or recreation facilities in the Al-Marashda region (Table 1).

**Table 1:** Opinions of Al-Marashda residents about the quality of life in their region

Opinions	Categories	Total = 184 n (%)
There is plenty of economic opportunity in Al-Marashda region.	Agree	45 (24)
	Disagree	121 (66)
	Natural	18 (10)
There are good and sufficient schools in the Al-Marashda region.	Agree	43 (23)
	Disagree	115 (63)
	Natural	26 (14)
“There is good healthcare in Al-Marashda region.” Consider the cost and quality, number of options, and availability of healthcare in the region.	Agree	97 (53)
	Disagree	40 (22)
	Natural	47 (26)
Easy access to healthcare facilities	Agree	147 (80)
	Disagree	11 (6)
	Natural	26 (14)
Low crime rate in Al-Marashda region/safe neighborhoods	Agree	137 (74)
	Disagree	14 (8)
	Natural	33 (18)
There is plenty of help for people during times of need in Al-Marashda region.	Agree	138 (75)
	Disagree	16 (9)
	Natural	30 (16)
There are parks and recreation in Al-Marashda region.	Agree	2 (1)
	Disagree	174 (95)
	Natural	8 (4)

When the interviewees were asked, “In your opinion, what do you think are the three most important “health problems” in our community in the Al-Marashda region?” The problems that most often have the greatest effect on overall community health were diabetes (74%, n= 137), high blood pressure (67%, n= 123), income/poverty (52%, n= 95), air pollution (41%, n= 75) and dental problems (39%, n= 71) (Table 2).

**Table 2:** The most important health problems in the Al-Marashda region were identified based on the primary data

Health Problems	Frequency (n = 184)	Percentage (95% CI)
Diabetes	137	74 (67-80)
High blood pressure	123	67 (59-73)
Low-income/poverty	95	52 (44-59)
Air pollution	75	41 (33-48)
Dental problems	71	39 (31-46)
Respiratory/lung disease	62	34 (27-41)
Cancers	46	25 (19-31)
Motor vehicle crash injuries	45	24 (18-31)
Aging problems (e.g., arthritis, hearing/vision loss, etc.)	39	21 (15-28)
Heart disease and stroke	37	20 (14-27)
Drug and alcohol abuse	25	14 (9-19)

When the interviewees were asked, “Below is a list of Environmental Risks that may be found in the Al-Marashda region, please select which you feel very dangerous for you,” the environmental risks selected most often were outdoor air pollution (68%, n= 126), waste disposal (63%, n= 116) and water pollution (41%, n= 75). When the interviewees were asked, “In your opinion, which of the following services needs the most improvement in your community?”, the services selected most often were availability of employment (66%; n= 121), road maintenance (52%; n= 95), animal control (51%; n= 93), better/more recreational facilities (parks, trails, community centers, etc.) (49%; n= 91) and drug and alcohol abuse prevention (27%; n= 50). According to this statement, “In the following list, what do you think are the three most important “risky behaviors" in our community?” The behaviors selected most often were drug abuse (40%; n= 74), lack of exercise (38%; n= 70), alcohol abuse (27%; n= 50), overweight (25%; n= 46) and poor eating habits (20%; n= 36).

After being told to consider access, availability, cost, quality, and options in health care in the Al-Marashda region, 52% (n= 96) of respondents answered “yes” with the statement “Are you satisfied with the health care system in the community?” When interviewees were asked “How would you rate the overall health of our community?”, 26% (n= 48), 58% (n= 107), and 16% (n= 29) of respondents selected healthy, somewhat healthy, and unhealthy, respectively. Almost half of the respondents were smokers (49%). When interviewees were asked “During a normal week, other than in your regular job, do you engage in any exercise activity that lasts at least a half an hour?”, most respondents (69%) answered this question with “No”.

Group discussions

Twenty-four individuals participated in three focus groups, and these groups identified cross-cutting themes in the following four domains: personal health, elements of a healthy community, and needed improvements in healthcare access. The participants focused on problems such as air pollution caused by the cement plant and gas plant in the Al-Marashda region and waste. Participants were concerned about the seventeen cancer cases that were reported in the past few years. Almost all the focus groups discussed the increase in the percentage of unemployment and poverty that impact community health. Some participants were concerned that residents with no or little income have difficulty accessing services and thus substituting emergency department care for primary care.

Secondary Data

In the region of Al-Marashda, with a population totaling 12,536 residents, the secondary data have been limited. The educational infrastructure is comprised of merely six primary schools in three buildings (Binary). Additionally, during the year 2023, an average of 1073 individuals sought medical care at the Al-Marashda Primary Health Care Center (PHCC), while 26 attended at the psychiatric unit of care. Moreover, the vaccine coverage of measles and measles, mumps, and rubella (MMR) stood at 94.8 and 121, respectively.

Determination of health priorities

The prioritization of community health needs was identified based on the primary data, group discussion, and secondary data. The 10 areas identified from the data as health priorities were diabetes and high blood pressure, poverty and unemployment, contamination, dental problems, respiratory/lung disease, cancer, motor vehicle crash injuries, educational aspects, drug and alcohol abuse, and physical activity.

## Discussion

Identifying unmet health needs in a population is a critical process for local authorities aiming to design suitable and efficient programs to address these needs. Failure to address these needs may result in implementing a top-down strategy in delivering healthcare services, which mirrors the perceptions of a select few rather than the actual needs of the population [14]. Therefore, it performed a CHA survey among Al-Marashda, an unrepresented region, to identify and prioritize health needs using the North Carolina Guide.

Concerning “health problems”, Al-Marashda residents believed that diabetes, high blood pressure, low income/poverty, and pollution were the most important health problems in their region. necessity to education in the early detection of hypertension and diabetes mellitus in primary health care centers. Detection of these diseases may be due to easy access to health care facilities, as approximately 80% of respondents agreed that "there is easy access to health care facilities". In the same context regarding health services, more than half of the respondents (53%) said, “There is good health care in the Al-Marashda area.” This opinion may be indicating the presence of an integrated health center for primary health care in addition to Al-Manathira General Hospital, which is approximately 5 km from the village and provides health services to the residents of the region.

In the Al-Marashda region, a majority of the participants indicated encountering constrained economic prospects. This phenomenon can be attributed to the predominantly rural and agricultural characteristics of the area, which may not facilitate the array of economic ventures and job opportunities commonly available in urban environments.

In terms of education, there are six governmental schools according to the Al-Najaf Health Directorate, while there are no private schools in the area at all. These six schools are all primary schools, with three schools for boys and three for girls [15]; therefore, this may explain why approximately half of the respondents had no formal education.

During the interviews and when the respondents were asked about environmental risks that may be found in their region, they said that outdoor air pollution accounted for 68%. The occurrence of outdoor air pollution in the Al-Marashda region is logical; this region is considered one of the areas surrounding the Cement Factory, which is in the Al-Manathera district and is considered one of the most important industrial factories in the Middle Euphrates region. Two types of pollutants are generated from cement factories: dust pollutants and gaseous pollutants, such as sulfur oxides (SOx), nitrogen oxide (NOx), carbon monoxide (CO), and hydrocarbons, which directly affect human health [16].

Concerning to social relations between the residents of this area, 74% of the respondents reported that they enjoy safe neighbors. Moreover, 75% of them declared that there is plenty of help for people during times of need, respectively. These good characteristics among the residents will, of course, reduce the level of crime, especially since the area is predominantly tribal and religious, such as the other Al Najaf regions and Iraq.

Regarding the entertainment aspect, the region lacks tourism and entertainment services completely, and this was reflected in the opinion of the citizens who said “disagree” about “There are Parks and Recreation in the Al-Marashda Region”. Additionally, 63% of the respondents reported that they suffer from waste disposal, and 41% suffer from water pollution. Together, these serious environmental problems reduce the standard of well-being in the Al-Marashda region.

Limitations of the study

During the time this survey was being conducted, the team faced some challenges. One of these challenges was the survey collection method, which may have introduced some bias into the data collection where data were collected from individuals who were at home throughout the day (for example, unemployed individuals), and this explains why the highest percentage of the sample was from the 10-19-year-old age group who were out of school. Another limitation is that the random construction of houses led to difficulty in selecting samples in a regular way. Lastly, it was not developed the community diagnosis document and the necessity for advocacy and support from public health authorities and the city governorate to implement appropriate interventions and address the prioritized problems.

## Conclusions

Based on the primary data, group discussions, and secondary data, several high-priority issues in the Al Marashda region have been identified. The high prevalence of NCDs, particularly diabetes and hypertension, is not unique to Al Marashda but reflects a broader trend observed throughout Iraq. However, specific to Al-Marashda are the low-income levels of residents and environmental concerns, such as air pollution from the Cement Factory. Furthermore, in the realm of education, the most significant issue is the lack of secondary schools in the region.

Efforts should be intensified to mitigate environmental pollutants and waste from the Cement Factory to protect residents. Collaboration with the Ministry of Education is essential to address the shortage of secondary schools in Al-Marashda, organize literacy courses, and motivate participation. Despite satisfactory health services according to residents, further development is needed to fill gaps and enhance therapeutic health and immunization campaigns. Health promotion should include increased health education campaigns, seminars, and volunteer involvement. Establishing small production projects can provide job opportunities for young people and reduce poverty levels. Additionally, conducting a CHA every three years will help gather crucial information on the population's health needs and concerns, facilitating better community health planning.

## References

[1] Placide M, Pierre NJ, Fidele N (2021). Community health needs assessment in urban communities in Kigali City in Rwanda: A cluster-randomized trial. J Public Health Int.

[2] Earle-Richardson Earle-Richardson, Melissa Scribani, Wyckoff L, Strogatz D, May J, Jenkins P (2015). Community views and public health priority setting: How do health department priorities, community views, and health indicator data compare?. Health Promot Pract.

[3] (2024). States CHAotU. Assessing and addressing community health needs. https://www.chausa.org/docs/default-source/general-files/cb_assessingaddressing-pdf.pdf?sfvrsn=4.

[4] Ravaghi H, Guisset AL, Elfeky S, Nasir N, Khani S, Ahmadnezhad E, Abdi Z (2023). A scoping review of community health needs and assets assessment: concepts, rationale, tools and uses. BMC Health Serv Res.

[5] Chavan KD, Giri PA, Rajurkar S (2019). Approaches for implementation and updation of community health needs assessment: An updated review. Int J Community Med Public Health.

[6] Glasgow RE, Vogt TM, Boles SM (1999). Evaluating the public health impact of health promotion interventions: The RE-AIM framework. Am J Public Health.

[7] Glasgow RE, Lichtenstein E, Marcus AC (2003). Why don't we see more translation of health promotion research to practice? Rethinking the efficacy-to-effectiveness transition. Am J Public Health.

[8] Braun KL, Nguyen TT, Tanjasiri SP (2012). Operationalization of community-based participatory research principles: Assessment of the national cancer institute's community network programs. Am J Public Health.

[9] Dina Khaled, H.L. H.L., Gwynne Zodrow, HEALTH NEEDS ASSESSMENT: SYRIA, IRAQ IRAQ, LEBANON LEBANON, EGYPT AND JORDAN RELIGIOUS AND ETHNIC MINORITY (2024). USAID: HEALTH NEEDS ASSESSMENT: SYRIA, IRAQ, LEBANON, EGYPT AND JORDAN RELIGIOUS AND ETHNIC MINORITY. https://www.nolostgeneration.org/media/4126/file/Health%20needs%20assessment:%20Syria,%20Iraq,%20Lebanon,%20Egypt,%20and%20Jordan%20Religious%20and%20Ethnic%20Minority%20.pdf.

[10] Reed JF, Fleming E (2014). Using community health needs assessments to improve population health. N C Med J.

[11] Ghazal NK, Hussein AK (2017). Assessment the irrigation and drainage networks in AL-Manathera district using GIS. J Kufa-Phys.

[12] Alrikabi NK, Almosherefawi OJ (2021). Reality analysis of the state of spatial distribution of green areas using geographic information systems (GIS)-The holy city of Najaf as a case study. IOP Conf Ser: Earth Environ Sci.

[13] Rahmani A, Asgarian A, Aligol M, Ahmadi Z, Mohammadbeigi A (2020). International License Creative Commons Attribution License 4.0 Community Assessment for Identifying and Prioritizing the Problems of Jamkaran Village in Qom. Province in.

[14] Wright J, Walley J (1998). Assessing health needs in developing countries. BMJ.

[15] Department PH (2023). Schools dataset. In: Directorate NH, editor. https://alnajafhealth.gov.iq/?s=Schools.

[16] Wahed FA, AL-Hakkak Z (2023). Study effect of cement dust exposure on health of workers at kufa cement factory. AIP Conf Proc.

